# [^18^F]-(2S,4R)4-Fluoroglutamine PET Imaging of Glutamine Metabolism in Murine Models of Hepatocellular Carcinoma (HCC)

**DOI:** 10.1155/2022/5185951

**Published:** 2022-07-25

**Authors:** Youngho Seo, Miranda C. Craig, Stephanie T. Murphy, Jinjin Feng, Xin Chen, Mariia Yuneva

**Affiliations:** ^1^Department of Radiology and Biomedical Imaging, University of California, San Francisco, CA, USA; ^2^Department of Nuclear Engineering, University of California, Berkeley, CA, USA; ^3^Department of Molecular and Cell Biology, University of California, Berkeley, CA, USA; ^4^Department of Bioengineering and Therapeutic Sciences, University of California, San Francisco, CA, USA; ^5^The University of Hawaii Cancer Center, Honolulu, HI, USA; ^6^The Francis Crick Institute, London, UK

## Abstract

**Purpose:**

Quantitative *in vivo* [^18^F]-(2S,4R)4-fluoroglutamine ([^18^F]4-FGln or more simply [^18^F]FGln) metabolic kinetic parameters are compared with activity levels of glutamine metabolism in different types of hepatocellular carcinoma (HCC).

**Methods:**

For this study, we used two transgenic mouse models of HCC induced by protooncogenes, MYC, and MET. Biochemical data have shown that tumors induced by MYC have increased levels of glutamine metabolism compared to those induced by MET. One-hour dynamic [^18^F]FGln PET data were acquired and reconstructed for fasted MYC mice (*n* = 11 tumors from 7 animals), fasted MET mice (*n* = 8 tumors from 6 animals), fasted FVBN controls (*n* = 8 normal liver regions from 6 animals), nonfasted MYC mice (*n* = 16 tumors from 6 animals), and nonfasted FVBN controls (*n* = 8 normal liver regions from 3 animals). The influx rate constants (*K*_1_) using the one-tissue compartment model were derived for each tumor with the left ventricular blood pool input function.

**Results:**

Influx rate constants were significantly higher for MYC tumors (*K*_1_ = 0.374 ± 0.133) than for MET tumors (*K*_1_ = 0.141 ± 0.058) under fasting conditions (*P* = 0.0002). Rate constants were also significantly lower for MET tumors (*K*_1_ = 0.141 ± 0.135) than normal livers (*K*_1_ = 0.332 ± 0.179) under fasting conditions (*P* = 0.0123). Fasting conditions tested for MYC tumors and normal livers did not result in any significant difference with *P* values > 0.005.

**Conclusion:**

Higher influx rate constants corresponded to elevated levels of glutamine metabolism as determined by biochemical assays. The data showed that there is a distinctive difference in glutamine metabolism between MYC and MET tumors. Our study has demonstrated the potential of [^18^F]FGln PET imaging as a tool to assess glutamine metabolism in HCC tumors *in vivo* with a caution that it may not be able to clearly distinguish HCC tumors from normal liver tissue.

## 1. Introduction

Altered metabolism has been identified as a primary hallmark of cancer cells [1, 2]. One of the major metabolic pathways studied has been glycolysis since the discovery of the Warburg effect (increased glucose consumption and lactate production by tumor tissues in comparison with normal tissues under normal oxygen conditions) [2, 3]. The development and implementation of glucose analog 2-deoxy-2[18F]fluoro-D-glucose ([^18^F]FDG) has allowed visualization of this increased glucose metabolism through PET imaging. However, glucose metabolism is only a facet of the metabolic reprogramming of cancer cells as it only accounts for the early stages of glycolysis. Lately, researchers are beginning to explore new noninvasive imaging strategies to investigate other aspects of cellular metabolism and expand metabolic imaging's arsenal.

Glutamine is one of the main areas of interest as among tumor cells' abnormally regulated metabolic pathways, and it corresponds to a high rate of glutaminolysis and a deregulated Krebs cycle [2, 4, 5]. Specifically, glutamine can serve as an alternative energy substrate to glucose providing mitochondrial 2-oxoglutarate. Glutamine is also one of the major sources for building carbon skeletons and for nitrogen metabolism and biosynthesis [5–7]. Cancerous cells have a high rate of glutamine metabolism, making visualization highly sought after. A glutamine analog, PET radiotracer ^18^F-(2S,4R)4-fluoroglutamine ([^18^F]FGln) was synthesized in 2011 [8]. [^18^F]FGln uses the same cellular transporters as glutamine and is minimally metabolized [8, 9].

[^18^F]FGln PET is attracting clinical attention and has been evaluated for visualization in the breast [10–13], pancreas [10, 11], renal [10, 11], neuroendocrine [11, 14], lung [10, 11], colon [10, 11, 15], lymphoma [10, 11], multiple myeloma [16], bile duct [10], and glioma tumors [10, 11, 17–20] as well brain tumors [10, 11, 16, 21] and brain metastasis [11, 21]. Pancreatic tumors were the only category in which [^18^F]FGln PET did not show prominent uptake [10]. Here, we report our study exploring [^18^F]FGln imaging in primary liver cancer.

This exploration is vital as liver cancer is the third leading cause of cancer-related deaths globally, resulting in 830,000 deaths in 2020 [22]. Hepatocellular carcinoma (HCC) is the most common subtype of liver cancer, corresponding to around 90% of cases [23]. Unlike all other common cancers, the death rate of HCC continues to increase yearly [23]. And HCC can only be diagnosed based on imaging features [24]. Early effective diagnosis of HCC is needed as, due to its absence, about 80% of patients are diagnosed with advanced HCC where the median overall survival time is 1–2 months [25, 26].

Here, we studied [^18^F]FGln uptake in two metabolically distinct types of murine HCC, induced by ectopic expression of either MYC [27] or MET [28] oncogenes. We have shown previously that MYC-induced liver tumors have increased glutamine catabolism in comparison with normal livers, which is associated with the increased expression of Gls1, kidney-specific isoform of glutaminase, and decreased expression of Gls2, a liver-specific isoform. Our results also showed that in contrast to MYC tumors, MET tumors have increased glutamine production from glucose [29]. These results as well as the results demonstrating the increased expression of a glutamine transporter ASCT2 in MYC but not in MET tumors [29] suggested potential differential uptake of [^18^F]FGln in MYC and MET liver tumors and the potential ability to differentiate between them using [^18^F]FGln PET imaging.

## 2. Materials and Methods

All mouse procedures, including housing, feeding, and monitoring, followed established standard operating procedures approved by the UCSF Institutional Animal Care and Use Committee (IACUC) and Laboratory Animal Resource Center (LARC).

### 2.1. [^18^F]FGln Radiosynthesis

[^18^F]-(2S,4R)4-Fluoroglutamine was synthesized following the procedures described in [8] at the radiochemistry facility at the University of California, San Francisco (UCSF). Radiosynthesis and quality control processes followed standard procedures of the institution.

### 2.2. Murine Models

We used 3 cohorts of mice: control (FVBN), HCC induced by MYC, and HCC induced by MET. MYC and MET tumors were generated as described previously [27, 28]. Briefly, *Tet-o-MYC* and *Tet-o-MET* mice of FVBN background were crossed to FVBN mice carrying the liver-specific promoter, *LAP-tTA* [30]. LAP-tTA/Tet-o-MYC mating pairs were kept on doxycycline (200 mg/kg doxy chow) to suppress transgene expression in embryos. Their progenies were maintained on doxycycline until 4 weeks of age. Doxycycline was then removed, and mice were followed for evidence of tumor formation. LAP-tTA/Tet-o-MET mating pairs were kept on a regular diet. Some of their progenies were placed on doxycycline, while the rest were left on a regular diet and eventually developed tumors. Fasting and nonfasting conditions were investigated in MYC tumors, not MET tumors. MET tumors were investigated prior to our hypothesis that fasting and nonfasting conditions would affect MYC tumor uptake of the radiotracer. We realized that fasting could be a factor for variation for our results in MYC tumors as the MYC oncogene and glutamine are both involved in metabolism. MYC plays a key role in the regulation of aerobic glycolysis unlike MET which is not known to be strongly related to glutamine metabolism.

**Figure 1:**
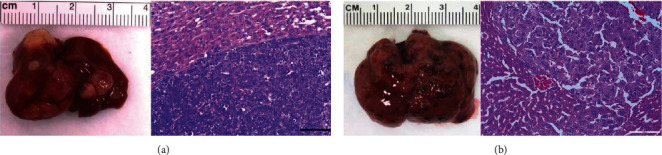
Liver tumor in MYC (a) and MET (b) models with accompanying H&E stains.

**Figure 2:**
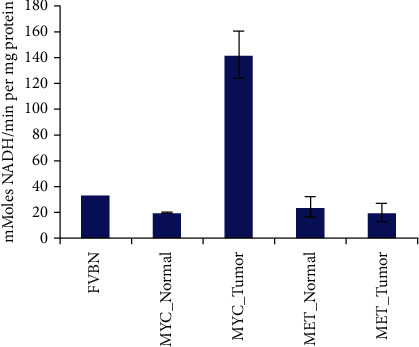
Glutaminase activity of HCC tumors (MYC tumor and MET tumor) and normal cells (MYC and MET) as well as normal livers from FVBN. Glutaminase activity measured in nMoles NADH/min per mg of the glutaminase protein present.

Figure 1 shows dissected HCC tumors induced by MYC and MET and hematoxylin and eosin (H&E) staining results to confirm the presence of these HCC tumors in murine models. We did not observe any metastatic lesions. MYC tumors have increased activity of phosphate-dependent glutaminase activity [31] in comparison with normal livers and MET tumors (Figure 2) consistent with their higher glutamine catabolism [29]. This supports our choice to compare [^18^F]FGln PET imaging of MET vs. MYC tumors.

Within these cohorts, we investigated [^18^F]FGln signal in tumors in comparison to the normal liver tissue under overnight fasting and nonfasting conditions.

### 2.3. Imaging Studies

Prior to [^18^F]FGln micro-PET/CT imaging studies, contrast-enhanced micro-CT was performed for the murine models of MYC and MET HCC tumors to confirm and anatomically define the presence of liver tumors *in vivo*. We used a custom-made mouse tail vein catheter comprised of a 28-gauge needle and a 100–150 mm-long polyethylene microtube (PE/1, Scientific Commodities, Inc., Lake Havasu City, AZ) to administer the lipid-emulsion iodinated contrast agent (Fenestra LC) 3-4 hours prior to micro-CT imaging. The placement of the catheter within the vein was confirmed by saline injection prior to the tracer injection. The scan parameters for *in vivo* micro-CT were 120 projections of continuous rotations to cover 220° with an X-ray tube operated at 80 kVp, 0.5 mA, and 175 ms exposure time per step. The CT data were reconstructed using a vendor-provided conebeam Feldkamp algorithm (COBRA, Exxim Computing Corporation, Pleasanton, CA).

For [^18^F]FGln micro-PET/CT imaging studies, we followed the procedure described in our previous work [31]. We administered 5.40-6.40 MBq of [^18^F]FGln intravenously using the catheter described above, and the PET data were acquired over 60 or 90 minutes. The absence of leakage and misinjection was verified for all micro-PET/CT scans by whole-body inspection of reconstructed PET images. Micro-CT imaging without contrast using the same acquisition and reconstruction techniques as for the separate contrast micro-CT as described earlier was performed, and CT-derived attenuation map was used for attenuation-corrected PET reconstruction using a three-dimensional ordered-subsets expectation-maximization with the maximum a posteriori (OSEM3D/MAP) algorithm provided by the scanner manufacturer. The dynamic multiframes (2 s × 15, 5 s × 6, 10 s × 6, 30 s × 4, 60 s × 6, and 300 s × 10 for 60 minutes or 300 s × 16 for 90 minutes) were constructed for kinetic modeling.

Animals were maintained under 1–2% isoflurane anesthesia during CT contrast and radiotracer administration and imaging sessions. A left ventricular blood pool volume of interest (VOI) was used as a blood input function for kinetic modeling.

### 2.4. Compartment Model and Visualization

Recent studies have demonstrated the utility of a one-compartment model to model [^18^F]FGln uptake in myeloma and breast xenografts [12, 13, 16, 18]. These studies have shown that in two-tissue compartment models the small value of *k*_3_ is difficult to estimate accurately and leads to spurious estimates of distribution volume of [^18^F]FGln, a one-tissue compartment model is favored and correlates well with changes in glutamine pool size [12]. We used a commercially available software package (Inveon Research Workplace, Siemens Medical Solutions USA, Inc., Malvern, PA) for all kinetic analyses included. The influx rate constants (*K*_1_) for [^18^F]FGln uptake in tumor tissue (MYC tumors: *n* = 11; MET tumors: *n* = 4) using a one-tissue compartment model were derived from dynamic PET data using the left ventricular (LV) blood pool as a blood input function. We used a small volume of interest in the blood pool, typically encompassing 2–3 voxels, well within the left ventricular chamber for the input function derivation in order to minimize the partial volume effect. The spherical tumor or liver VOIs were drawn (with a diameter of 2-3 mm) for target tissue in the compartment model. As far as they are distinguishable on contrast-enhanced CT, we drew multiple VOIs for tumors and normal liver tissues in the same animal. Finally, to show the general uptake difference visualization, we created parametric images of *K*_*i*_ ( = *K*_1_*k*_3_/(*k*_2_ + *k*_3_)) that is a net influx rate constant using a Patlak graphical model with the LV blood pool as a blood input function.

We used Amide (http://amide.sourceforge.net) for 2D and 3D visualizations of the contrast-enhanced micro-CT and [^18^F]FGln micro-PET/CT images.

### 2.5. Statistical Analysis

All kinetic parameters calculated were presented with the mean values and standard deviations. Statistical comparisons were based on unpaired *t*-test, and statistical significance was made for *P* < 0.05.

## 3. Results

### 3.1. Contrast-Enhanced Micro-CT

**Figure 3:**
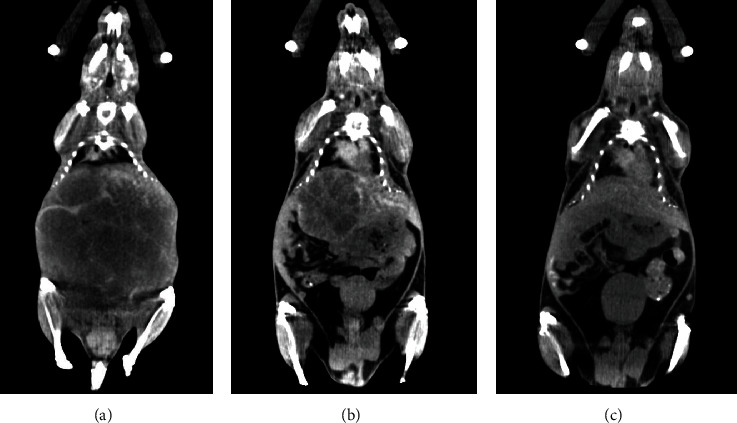
Example of micro-CT confirmation of MYC (a) and MET (b) tumors and normal liver (c).

HCC tumors were visualized by contrast-enhanced micro-CT (Figure 3), which was essential to locate the normal liver and tumors for our compartment model analysis.

### 3.2. Compartment Model Analysis

**Figure 4:**
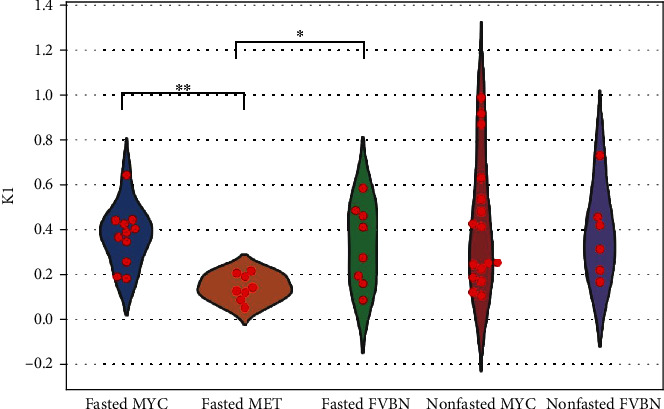
*K*_1_ values computed using one-tissue compartment model for fasted and nonfasted MYC tumors (*n* = 11 and *n* = 16 tumors, respectively), fasted MET tumors (*n* = 8), and fasted and nonfasted normal liver tissues (*n* = 8 and *n* = 6, respectively). ^∗∗^*P* = 0.0002 between fasted MYC and fasted MET and ^∗^*P* = 0.0123 between fasted MET tumors and fasted liver tissues. *P* = 0.5640 between fasted MYC and fasted normal liver, *P* = 0.7536 between nonfasted MYC and nonfasted normal liver, *P* = 0.5693 between fasted MYC and nonfasted MYC tumors, and *P* = 0.6039 between fasted and nonfasted normal liver tissues.

Under the fasting conditions influx rate constants (*K*_1_) using one-tissue compartment model were significantly lower for MET tumors than for MYC tumors (*K*_1_ = 0.141 ± 0.058 vs. *K*_1_ = 0.374 ± 0.133, *P* = 0.0002) and normal livers (*K*_1_ = 0.141 ± 0.135 vs. *K*_1_ = 0.332 ± 0.179, *P* = 0.0123; Figure 4). Influx rates for MYC tumors and normal livers were not different either in fasted or nonfasted conditions (*P* > 0.005). Interestingly, rate constants for nonfasted MYC tumors and normal livers had larger variabilities (i.e., higher standard deviation) than for fasted MYC tumors and normal livers (0.295 vs. 0.133 and 0.207 vs. 0.179, respectively). Our recent results demonstrated that although ^13^C-glutamine infusions resulted in the higher levels of ^13^C-glutamine-derived Krebs cycle intermediates in MYC tumors in comparison with adjacent normal livers, the levels of ^13^C-glutamine and ^13^C-glutamate were lower in the tumors than in the livers [32]. Lower glutamine and glutamate pools in tumors can be the result of their significantly higher catabolism into the Krebs cycle.

**Figure 5:**
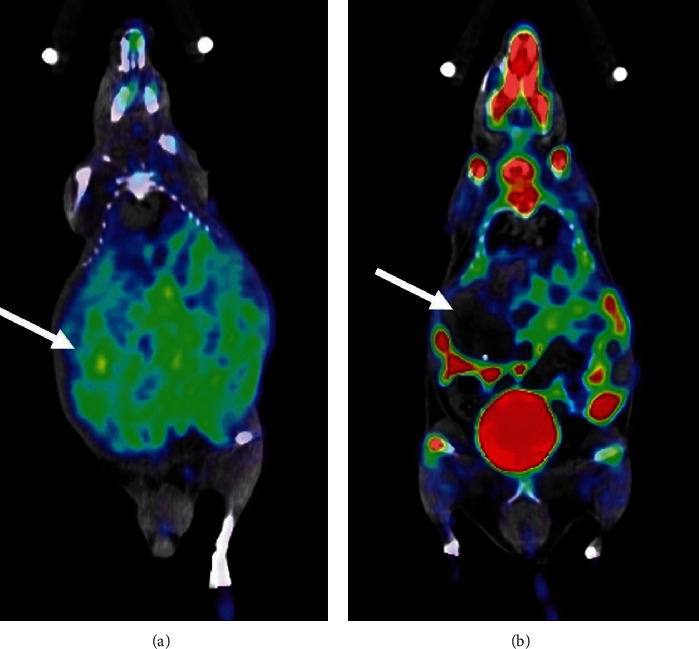
[^18^F]FGln parametric *K*_*i*_ images of a mouse with fasted MYC tumors (a) and a mouse with fasted MET tumors (b). Representative tumors are indicated with arrows, based on contrast-enhanced CT images (Figure 3).

In addition, we also generated parametric images of the net influx rate constant (*K*_*i*_ = *K*_1_*k*_3_/(*k*_2_ + *k*_3_)) using a Patlak graphical model to show the overall visual difference in [^18^F]FGln uptake in two types of tumors.  Figure 5 shows that there are much lower *K*_*i*_ voxels in MET tumors in comparison with higher levels of *K*_*i*_ voxels in MYC tumors.

## 4. Discussion

A limitation of our study was the small sample size; however, it should be noted that we achieved statistical significance for our primary goal of the imaging study, which was to differentiate MYC- and MET-induced HCC tumors under fasting conditions. An important finding was that the glutamine metabolism differences measured *in vivo*—higher influx rate constants and increased uptake—were reflective of the higher glutamine catabolism in MYC tumors compared to MET tumors [29]. Comparatively high signal in the normal liver can be reflecting accumulation of [^18^F]FGln catabolism due to its lower catabolism than in MYC tumors [32] and requires further research.

Another limitation of our study is that our input function does not account for the presence of potential metabolites as metabolite analysis has confirmed *in vivo* production of free ^18^F metabolite, hindering the analysis of tumors that are close to the bone [10]. However, overall, Zhou et al. reported that the contribution of labeled metabolites to the tumor PET signal in mice is small (≤10%) and unlikely to have a significant effect over image-derived metrics [13]. Due to our use of a using the left ventricular (LV) blood pool as a blood input function and a small volume of interest in the blood pool, typically encompassing 2–3 voxels, we believe this minimized this contribution even further. We also drew multiple VOIs for tumors and normal liver tissues in each animal (with a diameter of 2-3 mm) for target tissue in the compartment model. In vivo stability analysis by Grkovski et al. repeated the analysis of Zhou et al. [13] using a 3-compartment pharmacokinetic model with 2 input functions that account for nonspecific uptake of radiometabolites, and they found that the contribution to the total signal was about 10% (range, 0%–20%) [11]. The percentage signal from the third compartment was greater than 85% in bone tissue, as is expected because of accumulation of free ^18^F. However, due to our choice to use a one-tissue compartment model, this accumulation signal is avoided as the study by Grkovski et al. [11] indicates that the calculation of *K*_1_ and *k*_2_ is relatively robust, whereas *k*_3_ and *k*_4_ exhibit higher variance in models that assume free ^18^F does not significantly accumulate in tumors.

In order to explore the possible molecular mechanisms underlying our observation, we retrieved the data for major glutamine transporters as well as glutamine catabolism enzymes from the previously published microarray dataset for normal liver, MYC tumors, and MET tumors (Table 1) [29]. We can clearly see that multiple glutamine transporters are upregulated in MYC and MET HCC tissues, and for most of these transporters, MYC HCCs demonstrate a higher expression than that in MET HCC. A particular interest is the SLC7A6 gene, which encodes LAT2, a key glutamine transporter, which has been implicated in cancers [33]. SLC7A6 is found to be only regulated in MYC HCC, but not MET HCC, a very valuable result that suggests further studies to investigate this further and the clinical applications of therapies downregulating SLC7A6 as this clinical area is just beginning to emerge [34]. SLC7A6 has also been shown in murine models to regulate glutamine-dependent mTOR activation and decrease sensitivity in pancreatic cancer [33], leading to the hopes of using it as a therapeutic target for pancreatic cells. Our results suggest a similar pathway in HCC.

It would be of great interest for subsequent studies to investigate whether SLC7A6/LAT2 is the major glutamine transporter responsible for glutamine uptake in MYC HCC. To test this hypothesis, we could use CRISPR-Cas9-based gene editing to delete SLC7A6 in MYC-induced HCC. We can perform [^18^F]FGln imaging on the mice, and the results will provide key information about the role of this transporter in regulating glutamine update in MYC driven HCC.

In addition, there are 3 major glutamine catabolism enzymes: Gls and Gls2, which catabolize the deamination of glutamine into glutamate and ammonia, whereas Glul, which encodes GS, catabolizes the opposite reaction. It is important to note that Gls is strongly upregulated in MYC HCC, and Gls2, the major isoform in the liver, is mildly downregulated in MYC HCC. In contrast, Glul is strongly upregulated in MET HCC. The results indicate that MET HCC produces glutamine via endogenously generating this amino acid via glutamate. In contrast, MYC HCC actively utilizes glutamine during its progression [29]. The results are consistent with the increased glutamine uptake in MYC HCC.

For imaging [^18^F]FGln, we also found that rigorous fasting (over 12 hours) prior to the tracer administration is a preferred condition to evaluate the glutamine metabolism *in vivo* for measurement consistency. However, we also found that acute fasting did not have significant effect on [^18^F]FGln uptake in our data, suggesting further research into the effect of fasting (both acute and rigorous) on [^18^F]FGln uptake.

This study confirmed recent research on glutamine's efficacy as a radiotracer for PET imaging in cancer metabolism and presents the first analysis of [^18^F]FGln *in vivo* imaging of HCC. Our results align with the current literature on glutamine uptake that [^18^F]FGln uptake is described best by a one-compartment, reversible tissue model with no trapping.

## Figures and Tables

**Table 1:** Relative expression for major glutamine influx transporters and catabolism enzymes in the normal liver, MYC HCC, and MET HCC tissues based on the microarray studies.

	Gene name	Average Log2 expression		
		Normal liver	MYC HCC	MET HCC
Glutamine influx transporters	SLC1A5	6.09	**8.40 (** ^∗^ **)**	**7.18 (** ^∗^ **)**
	SLC6A14	4.93	4.75	4.70
	SLC6A19	5.35	5.80	5.50
	SLC7A5	6.68	**8.75 (** ^∗^ **)**	**8.20 (** ^∗^ **)**
	SLC7A6	6.32	**8.55 (** ^∗^ **)**	6.37
	SLC7A7	7.20	**8.23 (** ^∗^ **)**	**8.38 (** ^∗^ **)**
	SLC38A1	5.13	5.20	4.79
	SLC38A2	9.61	9.78	9.67
	SLC38A5	6.44	6.20	6.29
	SLC38A7	6.94	6.82	7.15
	SLC38A8	6.13	6.10	6.19

Glu catabolism emzymes	Gls	5.81	**9.07 (** ^∗^ **)**	6.08
	Gls2	8.42	*7.73 (* ^∗^)	*6.32 (* ^∗^)
	Glul	9.75	*8.39 (* ^∗^)	**11.9 (** ^∗^ **)**

^∗^ *p* < 0.05. Bold: upregulated in HCC. Italic: downregulated in HCC.

## Data Availability

Data are available from the corresponding author upon reasonable request.
